# Recent Advances and Future Perspectives on Heat and Mass Transfer Mechanisms Enhanced by Preformed Porous Media in Vacuum Freeze-Drying of Agricultural and Food Products

**DOI:** 10.3390/foods14172966

**Published:** 2025-08-25

**Authors:** Xinkang Hu, Bo Zhang, Xintong Du, Huanhuan Zhang, Tianwen Zhu, Shuang Zhang, Xinyi Yang, Zhenpeng Zhang, Tao Yang, Xu Wang, Chundu Wu

**Affiliations:** 1 School of Agricultural Engineering, Jiangsu University, Zhenjiang 212013, China; huxk@stmail.ujs.edu.cn (X.H.); 2111916002@stmail.ujs.edu.cn (H.Z.); 2212316020@stmail.ujs.edu.cn (X.Y.); 2212316063@stmail.ujs.edu.cn (Z.Z.); 2212316058@stmail.ujs.edu.cn (T.Y.); 2112316032@stmail.ujs.edu.cn (X.W.); 2 Key Laboratory for Theory and Technology of Intelligent Agricultural Machinery and Equipment, Jiangsu University, Zhenjiang 212013, China; 3 Jiangsu Province and Education Ministry Co-Sponsored Synergistic Innovation Center of Modern Agricultural Equipment, Jiangsu University, Zhenjiang 212013, China; 4 School of the Environment and Safety Engineering, Jiangsu University, Zhenjiang 212013, China; 2112209003@stmail.ujs.edu.cn (X.D.); shua.zhang@stmail.ujs.edu.cn (S.Z.); 5 School of Computer Science and Communication Engineering, Jiangsu University, Zhenjiang 212013, China; 2222308079@stmail.ujs.edu.cn

**Keywords:** food processing, vacuum freeze-drying, preformed porous media, heat and mass transfer, porous structures, multi-field coupling

## Abstract

Preformed porous media (PPM) technology has emerged as a transformative approach to enhance heat and mass transfer in vacuum freeze-drying (VFD) of agricultural and food products. This review systematically analyzes recent advances in PPM research, with particular focus on spray freeze-drying (SFD) as the dominant technique for precision pore architecture control. Empirical studies confirm PPM’s efficacy: drying time reductions of 20–50% versus conventional VFD while improving product quality (e.g., 15% higher ginsenoside retention in ginseng, 90% enzyme activity preservation). Key innovations include gradient porous structures and multi-technology coupling strategies that fundamentally alter transfer mechanisms through: resistance mitigation via interconnected macropores (50–500 μm, 40–90% porosity), pseudo-convection effects enabling 30% faster vapor removal, and radiation enhancement boosting absorption by 40–60% and penetration depth 2–3 times. While inherent VFD limitations (e.g., low thermal conductivity) persist, we identify PPM-specific bottlenecks: precision regulation of pore structures (<5% size deviation), scalable fabrication of gradient architectures, synergy mechanisms in multi-field coupling (e.g., microwave-PPM interactions). The most promising advancements include 3D-printed gradient pores for customized transfer paths, intelligent monitoring-feedback systems, and multiscale modeling bridging pore-scale physics to macroscale kinetics. This review provides both a critical assessment of current progress and a forward-looking perspective to guide future research and industrial adoption of PPM-enhanced VFD.

## 1. Introduction

Vacuum freeze drying (VFD) is an advanced dehydration technology that has attracted considerable attention in agricultural and food processing due to its distinctive advantages [1,2]. Operating under low temperature and vacuum conditions, VFD minimizes thermal degradation and oxidative damage to heat-sensitive components, thereby effectively preserving the original color, aroma, flavor, texture, and nutritional quality of food products [3,4]. During the process, Moisture sublimates directly in the form of ice, avoiding the influence of liquid water, so that the product almost does not shrink, forming a loose porous sponge-like structure, excellent rehydration, and almost unchanged volume shape [5]. For instance, freeze-dried fruit powders retain the vivid color and bioactive compounds of the original fruit; freeze-dried mango powder closely resembles mango puree in color and exhibits outstanding rehydration performance [6]. Similarly, freeze-drying enhances the retention of functional compounds such as total phenolics and flavonoids in passion fruit byproducts, improving their nutritional value [7]. VFD-treated products—such as fruits, vegetables, and soups—rehydrate rapidly and deliver a near-fresh sensory experience, which is difficult to achieve using conventional techniques like hot-air drying [8,9,10,11]. Moreover, the low-temperature vacuum environment suppresses microbial growth and enzymatic activity, significantly extending shelf life [12]. As a result, VFD is widely applied in the processing of high-value foods, including instant coffee, functional fruit and vegetable powders, and porcine or bovine bile powder [13,14,15], as well as in the preservation of bioactive substances such as microbial cultures, enzyme preparations, and traditional Chinese medicinal herbs [16,17,18].

Despite the unparalleled advantages of vacuum freeze drying (VFD) in preserving product quality, its high energy consumption and prolonged drying time remain major barriers to large-scale industrial application [19]. It has been reported that the energy consumption of VFD is approximately 4 to 10 times higher than that of hot-air drying, making it one of the most expensive dehydration operations in the food industry [20]. A typical food freeze-drying cycle often exceeds 20 h, and for thick samples, it may extend over several days [21]. The root cause of this limitation lies in the inherently low efficiency of heat transfer (HT) and mass transfer (MT) during the VFD process, particularly during the later stage of drying, known as the desorption or secondary drying phase, where resistance to transfer significantly increases [22]. In terms of heat transfer, convective heat exchange becomes negligible under high vacuum, leaving conduction through the shelf and radiation from the chamber walls as the primary heat sources [23]. However, the thermal contact resistance between the material and the metal shelf is substantial, and as drying proceeds, the growing porous dried layer (dry layer) exhibits extremely low thermal conductivity—typically around 0.1 W/m·K, which is an order of magnitude lower than that of ice—becoming a major bottleneck for heat delivery to the internal sublimation interface. As a result, the interface temperature can be several tens of degrees lower than the shelf temperature [24]. Moreover, to prevent structural collapse (occurring above Tg’ ≈ −40 °C to −12 °C depending on composition [3,19]) or ice melting (at >−0.5 °C under vacuum [25]), shelf temperatures are typically constrained to −40 °C to −10 °C. This conservatism limits heat flux to <0.5 kW/m^2^, directly suppressing sublimation rates. On the mass transfer side, the water vapor generated from ice sublimation must diffuse through the increasingly thick and porous dry layer before being removed by the vacuum system [26]. The pore network formed during conventional freezing is typically fine and tortuous, and vapor transport occurs mainly via molecular diffusion and Knudsen (slip) flow, both of which exhibit low effective mass transfer coefficients. As drying progresses, the sublimation front retreats toward the material core, extending the vapor diffusion path through the dried layer to the external surface [27]. Due to the lack of convection drive in a vacuum, it is difficult to quickly remove a large amount of water vapor by relying only on a small partial pressure gradient to promote diffusion. The porous structure of the drying layer hinders the gas flow, which may lead to the accumulation of water vapor, an increase in local pressure, and even lead to complex phenomena such as ‘secondary evaporation condensation’, affecting the drying stability. Collectively, these limitations result in a significantly slower drying rate compared to conventional methods, thereby constraining the productivity and economic viability of VFD [28].

To address the low efficiency of vacuum freeze drying (VFD), researchers have proposed a variety of enhancement strategies. These include: (1) Optimization of process parameters, such as adjusting shelf temperature and chamber pressure, implementing stepwise heating protocols, and employing real-time endpoint detection to improve process control [2]; (2) Pretreatment of raw materials, including size reduction (e.g., slicing or dicing), surface modification (e.g., peeling or perforation), osmotic dehydration, and tissue structural modification through techniques such as pulsed electric fields (PEF), high hydrostatic pressure (HHP), or ultrasound, which can reduce internal resistance to drying [5,8,9]; and (3) Assistance from external energy fields, including ultrasound (to expand micropores via cavitation and induce micro-vibrations), infrared radiation (to provide shallow-penetration surface heating), microwave and radio-frequency heating (to generate internal heating), and electric field treatments, all of which aim to accelerate ice sublimation and water vapor transport [13,14]. While these approaches demonstrate efficacy—for instance, microwave-assisted VFD reduces SEC to <12–15 kWh/kg versus 36–42 kWh/kg for conventional VFD [29]—they face limitations like localized overheating. In contrast, PPM achieves 20–70% SEC reduction by structurally accelerating drying without intensive external energy, minimizing quality risks. Potential drawbacks include the risk of localized overheating and thermal degradation of sensitive bioactive compounds (particularly with microwave or infrared radiation), increased equipment complexity, high operational costs, and limited applicability to certain types of materials.

In recent years, PPM has emerged as an innovative self-enhancement strategy based on the internal structural engineering of materials, demonstrating significant potential for improving vacuum freeze drying (VFD) efficiency [30]. The central concept of PPM is to pre-establish a controllable, highly permeable, and interconnected pore network within the material prior to drying, using specific physical or chemical methods such as spray coating or spray freeze-drying, foaming, mechanical perforation, or the introduction of removable templates [31]. PPM introduces engineered macropores (typically 50–500 μm), which are 5–50 times larger than natural ice-templated pores (1–20 μm). This dimensional shift transforms vapor transport from diffusion-limited Knudsen flow (effective diffusion coefficient D_eff_ < 10^−6^ m^2^/s) to convection-capable regimes (D_eff_ > 10^−5^ m^2^/s), reducing pressure gradients by >30% [32]. Early theoretical models predicted, and subsequent experiments—such as the work of Thomik et al. confirmed, that the presence of initial porosity could shift the sublimation pattern from a planar front to a more distributed or volumetric mode, thereby accelerating the drying rate by 20–50% or more [27]. Additionally, PPM induces three synergistic effects: (i) Front Dispersion: Initial pores enable sublimation at multiple distributed sites (volumetric drying), replacing a single planar front; (ii) Thermal Path Optimization: This reduces the maximum heat penetration distance (L_eff_↓) while pore walls provide additional solid-phase conduction bridges; (iii) Energy Field Enhancement: Macro-pores act as waveguides for radiation/microwaves, while pore curvature concentrates electromagnetic fields at edges, creating localized absorption zones [33]. Collectively, these effects accelerate drying without external energy escalation. All in all, PPM is the concept of shortening time by structural optimization. reducing internal process resistance through favorable structural preconditioning rather than continuous external energy input, in contrast to field-assisted techniques. This intrinsic enhancement offers several advantages: it does not markedly increase energy consumption, exerts minimal impact on product quality, is synergistically compatible with other intensification methods (e.g., microwave-assisted drying), and is broadly applicable to materials in liquid, slurry, or solid forms.

**Figure 1:**
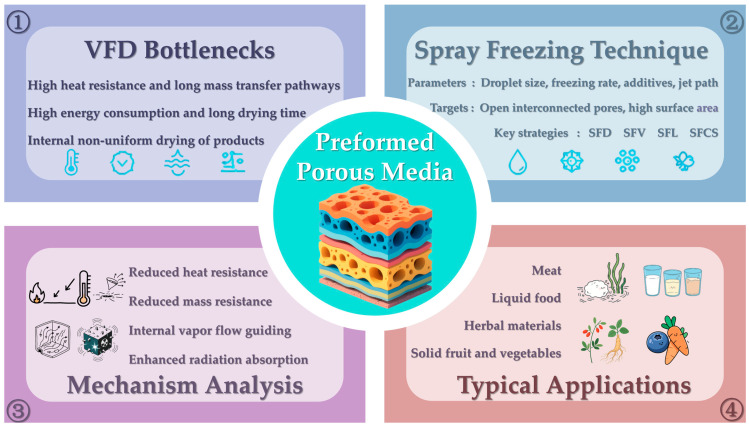
Multi-mechanism enhancement of vacuum freeze-drying strengthened by preformed porous media.

This review focuses on two major categories within agricultural and food systems: (1) high-value liquid products (near 0% initial porosity; viscous matrices limit ice crystal growth during freezing), such as milk, whey, coffee extracts, tea infusions, fruit juices and purées, plant- and animal-derived protein extracts (e.g., porcine and bovine bile extracts), fermentation broths (e.g., yeast and lactic acid bacteria cultures), and enzyme solutions; and (2) solid agricultural and food materials (15–25% porosity from cell walls and vascular bundles, yet wax cuticles/septa create diffusion barriers), including fruits (e.g., strawberries, mangoes, blueberries), vegetables (e.g., carrots, bell peppers, kelp), meats, aquatic products, and medicinal-food homologous materials (e.g., ginseng, goji berries, mushrooms). Particular emphasis is placed on the use of spray-based techniques, especially spray freeze-drying (SFD), to engineer porous structures prior to drying. Various SFD configurations are discussed—including atomization above cryogenic liquids, into cold gaseous media, into liquid nitrogen, or onto cryogenic metal surfaces—as key methods for structural modification. The influence of critical process parameters such as droplet size, freezing rate, and additives on pore morphology is analyzed, along with the associated application challenges. A systematic review and in-depth analysis are presented on recent advances in the use of PPM to enhance heat transfer (HT) and mass transfer (MT) mechanisms during vacuum freeze drying (VFD) of agricultural and food products. For solid agricultural products (e.g., fruits, vegetables, meats), PPM implementation requires distinct pore-formation strategies: Mechanical methods: Laser micro-drilling (50–200 μm pores), needle perforation; Physical fields: Pulsed electric fields (PEF), high hydrostatic pressure (HHP), or ultrasound to induce micro-channels; Cryo-structuring: Controlled freezing protocols to engineer ice-templated macropores. These techniques—detailed in Section 4.2—enable vapor pathway creation while preserving tissue integrity. The discussion highlights how PPM overcomes traditional VFD limitations by structurally modifying the internal matrix of the material, elucidating its working principles, key influencing factors, and demonstrating performance across various systems. Multi-mechanism enhancement of vacuum freeze-drying strengthened by preformed porous media is shown in Figure 1. The future outlook on PPM-driven VFD intensification is also discussed, offering novel insights and a promising technological pathway for innovation in food engineering and agricultural product processing.

## 2. Spray-Based Techniques for Constructing Porous Structures: Methods and Applications

The formation of PPM requires specific treatments either prior to or during the freezing process. Among the key techniques for introducing porosity in food and biobased materials, spray-based methods—including spray freezing, spray-induced nucleation, and coating-based freezing—have shown particular promise. The fundamental concept involves atomizing a liquid material into a cryogenic medium, where rapid cooling leads to the formation of frozen particles or films with defined microstructures, resulting in an initial material characterized by high surface area and porosity [34,35]. Spray techniques enable precise control over droplet size, freezing rate, and pore morphology, and are considered one of the most effective approaches for achieving PPM structures [36,37]. This section provides a comprehensive overview of the main types of spray-based pore-forming technologies, relevant process parameters, their effects on pore structure, and the current challenges associated with their application.

### 2.1. Types of Spray Freeze-Drying (SFD) Technologies

Spray freeze-drying (SFD) is a representative spray-based technique for pore-structure formation, in which a liquid feed is atomized into fine droplets, rapidly frozen, and subsequently dried via sublimation directly from the frozen particles [38]. Depending on the type of freezing medium used to solidify the atomized droplets, various SFD configurations have been developed.

#### 2.1.1. Spray Freezing into Vapor over Liquid (SFV/L)

In this approach, a layer of cold gas forms above the surface of a cryogenic liquid, such as liquid nitrogen, creating a low-temperature vapor phase. When atomized droplets of the feed material are sprayed into this gaseous layer, they undergo partial cooling before falling into the underlying cryogenic liquid, where complete solidification occurs. As partial freezing takes place during the gas-phase transit, this method reduces droplet splashing and coalescence upon contact with the liquid nitrogen [39]. However, the short residence time in the cold gas layer may result in some droplets entering the cryogenic liquid before fully freezing. The technique offers the advantage of simplified particle collection, but it also presents challenges such as cryogen evaporation and the need for efficient cryogen recovery systems.

#### 2.1.2. Spray Freezing into Vapor (SFV)

In spray freezing into a cold vapor (SFV), atomized droplets are introduced into a stream of cold gas—typically refrigerated nitrogen or supercritical CO_2_—where they gradually freeze during their descent and are subsequently collected on a chilled surface or filter mesh. This method eliminates the need for liquid nitrogen, offering operational convenience and simplified particle collection. However, due to the limited contact time between the droplets and the cold gas, incomplete freezing may occur before the droplets reach the collection surface, resulting in irregular particle morphology [40].

#### 2.1.3. Spray Freezing into Liquid (SFL)

In spray freezing into liquid (SFL), atomized droplets are directly injected into a cryogenic liquid such as liquid nitrogen, where they undergo instantaneous solidification due to the extreme temperature gradient. This process enables ultrafast freezing rates—up to 10^3^ K/s for ~10 µm droplets in liquid nitrogen [41]—resulting in the formation of very fine ice crystals and highly porous structures. However, high-pressure atomization is required to overcome the resistance of the dense cryogenic medium, and precautions must be taken to prevent nozzle blockage caused by freezing and ice accumulation [42].

#### 2.1.4. Spray Freezing onto Cooling Surface (SFCS)

In spray freezing onto a cryogenic surface (SFCS), atomized droplets are directly sprayed onto a solid plate that has been pre-cooled to subzero temperatures. Upon impact, the droplets spread into thin films and rapidly freeze into sheet-like structures [43]. These frozen films are then mechanically scraped from the surface using a blade and subsequently subjected to vacuum drying. Unlike other spray freezing methods that yield particles, SFCS produces thin flakes, whose pore structures are largely governed by the solidification dynamics of the liquid film. This technique enables more uniform freezing but requires additional scraping steps [44].

Each of the aforementioned spray freezing techniques offers distinct advantages and limitations. Overall, spray freezing into liquid (SFL) achieves the highest freezing rate and produces the smallest particles with the finest pore structures, but it demands high energy input and complex operation. In contrast, spray freezing into vapor (SFV) is simpler and easier to implement but involves a trade-off between complete droplet solidification and preservation of particle morphology. Hybrid gas-liquid systems (SFV/L) and SFCS provide intermediate solutions that balance the strengths and weaknesses of the former two approaches. The choice of method should be tailored to the material properties and equipment constraints. For instance, SFCS is well-suited for high-sugar, high-viscosity liquids to produce uniform porous freeze-dried sheets for instant beverage applications, while SFL is preferred for dairy-based products and probiotics, enabling the production of fine freeze-dried powders with improved dispersibility and storage stability. Regardless of the specific method, all spray freezing techniques share a common mechanism: by reducing droplet size and enabling ultrafast freezing, they promote the development of highly porous microstructures within the material. This significantly increases the number of mass transfer pathways and shortens their effective lengths, thereby creating favorable conditions for accelerated freeze-drying [45]. Main types and comparison of programmable spray technologies for porous structure formation are shown in Table 1.

### 2.2. Process Parameters and Pore Structure Control

The effectiveness of spray-based porous structure formation is influenced by multiple process parameters, including droplet size, freezing rate, and solution properties. These factors collectively determine the microstructural characteristics during solidification and, consequently, the resulting pore morphology [46]. The primary influencing parameters are summarized as follows.

#### 2.2.1. Droplet Size and Initial Concentration

Smaller droplets (e.g., 1–50 μm) enable ultra-rapid freezing, yielding fine ice crystals and high porosity (70–90%). Conversely, larger droplets (200–1000 μm) develop thermal gradients, forming dendritic ice and lower porosity (40–60%). Higher solute concentrations (e.g., >30% solids) promote segregation during freezing, reducing porosity to 30–50%, while dilute solutions (<10% solids) achieve >80% porosity—though mechanical stability may suffer [47,48]. In addition, the initial solid content of the solution plays a critical role: higher concentrations promote solute segregation during freezing, which leads to thicker pore walls and reduced overall porosity. Reducing solid concentration to 5–10% yields high porosity (80–95%), ideal for rapid drying, but risks structural fragility (e.g., collapse stress <5 kPa). Medium concentrations (10–30% solids) achieve balanced porosity (60–80%) with adequate strength (>15 kPa). Concentrated solutions (>30% solids) suppress porosity to 30–50%, enhancing stability but prolonging drying. Optimization targets 20 ± 5% solids for most applications to maximize porosity without compromising handling [47]. Therefore, optimization of solution concentration prior to spraying is essential to balance pore architecture with structural stability.

#### 2.2.2. Freezing Medium Temperature and Freezing Rate

Liquid nitrogen freezing (−196 °C, rate >10^3^ K/s) suppresses crystal growth, producing microporous structures (85–95% porosity). In contrast, slower freezing (e.g., −40 °C ethanol bath, rate ~10^1^ K/s) generates larger pores but reduces porosity to 50–70%. Optimized freezing protocols balance pore connectivity (requiring >60% porosity) with structural integrity [49]. Rapid freezing also reduces solute segregation and inhibits ice recrystallization, yielding structures that are closer to amorphous. For instance, in pharmaceutical freeze-drying, spray freezing with liquid nitrogen has been used to produce X-ray amorphous porous particles. However, too fast freezing may produce a large number of submicron pores. Although drying is accelerated, too fine pores may affect the rehydration performance. On the contrary, slower freezing will generate large pores, which are conducive to mass transfer, but too large pores (>100 μm) will affect the appearance and taste of the product. Therefore, freezing rate is often controlled by adjusting the temperature and flow rate of the cryogenic medium to obtain the desired pore size distribution. Coffee extract was spray-frozen into pre-cooled ethanol baths at different temperatures, and particles frozen at –78 °C exhibited pore sizes approximately 30% smaller than those formed at –40 °C, while achieving nearly twice the drying rate—highlighting the benefit of low-temperature rapid freezing for improving drying efficiency [29].

#### 2.2.3. Additives and Nucleation Control

In some cases, ice nucleating agents or seed crystals are added to the formulation to induce specific pore structures. For example, adding 0.1–1.0 wt% nucleating agents (e.g., silica microparticles) increases pore density, elevating porosity by 15–25% versus untreated controls. Foaming agents (e.g., 0.5% Tween 80) create dual-pore systems, boosting porosity to 75–90% by incorporating gas-derived macropores [50]. Gradient freezing further tunes porosity radially (e.g., surface: 60% porosity with 10 μm pores → core: 80% porosity with 50 μm pores). Additionally, control over the nucleation temperature gradient can be used to modulate pore size distribution. For instance, stepwise reduction of the environmental temperature during spraying can generate a radial gradient in ice crystal size, resulting in hierarchical pore structures that become progressively larger from the surface to the core.

Ice nucleating agents or seed crystals are sometimes added to induce the formation of specific pore architectures. For instance, the inclusion of a small amount of insoluble solid microparticles as nucleation cores can increase the number of ice crystals and reduce their size, thereby enhancing pore density [50]. Alternatively, foaming agents can be incorporated into the spray solution to generate microbubbles during atomization, which are then trapped and frozen within the droplets. This results in a dual-pore system comprising ice crystal-derived pores and gas bubble-derived pores. These bubble-originated pores act as preformed open channels that significantly facilitate water vapor escape during the initial drying stage. Moreover, controlling the thermal gradient of nucleation can also modulate pore size distribution. For example, stepwise reduction of the spraying environment temperature can induce a radial gradient in ice crystal size, forming pore structures that gradually increase from the surface to the core. By adjusting these parameters, researchers can precisely tailor the pore architecture within a defined range. Niwa employed ultrasonic atomization with a four-fluid nozzle combined with liquid nitrogen freezing to produce porous particles with an average size of only 5 µm and uniform submicron internal pores [51]. Thomas used controlled cryogenic plate temperatures to fabricate freeze-dried ceramic foams exhibiting a gradient transition from fine surface pores to larger interior ones [52]. In food applications, controlling pore structure is critical not only for drying efficiency but also for the texture and rehydration behavior of the final product. For example, spray freeze-dried milk powders with overly large pores may dissolve quickly but yield a fluffy, loosely packed reconstituted form; conversely, excessively small pores can impede rehydration. Therefore, pore size must be optimized based on the intended application. Additionally, spray conditions influence particle morphology—such as whether particles are solid, hollow, or spherical—and affect powder flowability, all of which must be carefully considered in formulation and process design. Summary of the relationship between process parameter control and pore structure regulation in spray freeze drying is as follows (Table 2).

### 2.3. Structural Design Strategies and Application Challenges

#### 2.3.1. Structural–Functional Integration

Spray freezing not only facilitates the formation of porous structures but also enables the incorporation of specific functional attributes through formulation and process design. In functional food applications, spray freezing can be used to encapsulate probiotics, vitamins, and other bioactive compounds within a porous matrix, resulting in freeze-dried functional microparticles that are both highly soluble and protective of sensitive ingredients. This approach achieves a seamless integration of structural engineering and functional loading [54]. Similarly, spray freezing has been employed in the fabrication of orally disintegrating tablets (ODTs), where the porous structure ensures rapid disintegration upon contact with water, while carefully selected excipients provide the mechanical strength necessary for tablet integrity [55]. This structural–functional integration has been successfully applied in pharmaceutical formulations—for example, in Zydis^®^ fast-dissolving tablets, which are manufactured by spray freezing followed by lyophilization. Such design principles could be extended to food systems in the future, enabling the development of instant, reconstitutable functional foods with tailored nutrient delivery and flavor release characteristics.

#### 2.3.2. Gradient Pore Structures

In certain applications, it is desirable for different regions of a product to exhibit distinct pore sizes—for example, smaller pores on the outer surface to enhance mechanical strength and larger pores in the core to promote rapid rehydration. Such gradient pore structures can be achieved by modulating freezing conditions or through layer-by-layer spray deposition. In some cases, materials are first sprayed onto a cryogenic plate to form a thin film with fine pores, followed by continued deposition to produce a coarser porous core. This results in a freeze-dried body with a dense outer shell and a loosely structured interior, providing external protection while enabling rapid dissolution of the core. A similar gradient design strategy is employed in the fabrication of scaffolds for tissue engineering, where freeze-drying is used to create pore sizes that increase from the surface to the interior, mimicking natural tissue transitions [53]. Precise control of time–temperature protocols is essential for constructing such gradient architectures. Although this approach has been extensively studied in biomedical applications, it remains underexplored in food freeze-drying but holds significant potential for future development.

#### 2.3.3. Integrated Quality Control

While accelerating the drying process is a major objective, it is equally critical to ensure overall product quality, including appearance, texture, nutritional value, and safety. Spray freeze-drying (SFD) may introduce new challenges, such as the formation of ultrafine powders with increased hygroscopicity and powdering during reconstitution. Rapid freezing may also induce amorphous structures, which can compromise storage stability; in some cases, a controlled degree of crystallinity is required to prevent moisture-induced caking in freeze-dried food powders. Therefore, pore structure optimization must be accompanied by careful monitoring and adjustment of these quality-related properties. For instance, the addition of stabilizers such as polysaccharides or polyols to the spray solution can enhance the stability of the dried powder [56], while coating or granulation techniques may be employed to improve powder flowability. The impact of pore architecture on sensory properties should also be evaluated—for example, excessively large pores in freeze-dried fruit pieces may lead to undesirable chewiness or fragility. When applying PPM strategies, these quality attributes must be holistically considered to achieve a balance between process efficiency and product integrity.

**Figure 2:**
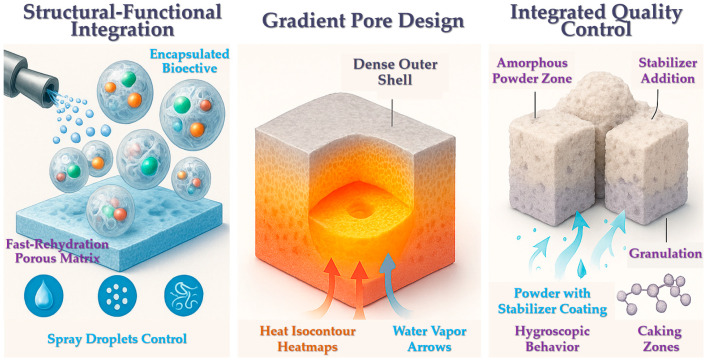
PPM structure design strategy and application challenges. (This original conceptual figure was created by the authors using 3D Max to generate the base 3D schematic elements, followed by refinement, labeling, and layout adjustments in Adobe Illustrator 2024. The figure is illustrative only and not derived from numerical simulation data).

In summary, spray-based technologies offer a flexible and effective approach for constructing porous structures in freeze-dried materials. Various spray freezing techniques can be tailored to generate pores of different sizes and morphologies, thereby enhancing heat and mass transfer during freeze drying. However, challenges such as process scale-up and quality control must be addressed before widespread industrial implementation can be achieved. With ongoing advances in process optimization and structural design, spray-assisted pore engineering is expected to play an increasingly important role in the intensification of freeze-drying processes. The design strategy and application challenges of the PPM structure are shown in Figure 2.

## 3. Mechanistic Analysis of Heat and Mass Transfer Enhancement by Porous Media

The ability of PPM to significantly enhance the freeze-drying process lies in its fundamental alteration of internal heat and mass transfer pathways within the material. This section presents a mechanistic analysis of how PPM reduces transport resistance, facilitates vapor convection, enhances radiative heat transfer, and improves conductive heat pathways during freeze drying. Each mechanism is discussed in detail from the perspective of transport phenomena, with reference to recent studies that illustrate the underlying principles and observed effects.

### 3.1. Reduction of Heat and Mass Transfer Resistance (Pore-Induced Resistance Mitigation)

#### 3.1.1. Reduction of Water Vapor Diffusion Resistance

One of the most immediate effects of PPM is the significant enhancement of permeability in the dried layer, which in turn reduces resistance to water vapor diffusion. In conventional freeze drying, the dried layer typically exhibits low porosity and small pore sizes, where water vapor transport is primarily governed by a slow Knudsen-type diffusion mechanism [57]. With the introduction of PPM, a certain volumetric fraction of larger, pre-existing pores is embedded within the matrix, substantially increasing the effective porosity. Model calculations by Ayaulym et al. showed that increasing pore diameter in the dried layer from 5 µm to 50 µm resulted in more than a one-order-of-magnitude increase in the effective diffusion coefficient, indicating a dramatic reduction in vapor diffusion resistance [58]. Experimental work by Wang et al. further confirmed that samples containing 10% initial porosity exhibited approximately a 30% reduction in vapor pressure gradient during the later drying stages compared to non-porous controls, demonstrating the substantial drag-reducing effect of embedded pores [59]. Moreover, pore connectivity is critical to achieving these benefits: the presence of continuous, straight channels formed by PPM provides direct “fast tracks” for vapor escape, allowing water vapor to reach the surface with minimal hindrance. In contrast, disconnected or isolated pores contribute little to mass transfer enhancement. Comparative studies of different pore morphologies revealed a clear trend in drying rates: interconnected macropores > closed isolated pores > non-porous structures, confirming that only open and continuous pore networks can fully realize the resistance-reduction effect of PPM [27,59]. This trend is consistent with experimental and modeling observations showing that larger, continuous pores markedly increase effective diffusion coefficients and reduce vapor-pressure gradients [58,59], while pore-structure–dependent drying kinetics and sublimation-front patterns have been directly compared by Thomik et al. [27].

#### 3.1.2. Thermal Conduction Dynamics in Porous Media: Resistance Mitigation vs. Gas Trapping Effects

While introducing pores reduces bulk thermal conductivity (κ) due to gas-filled voids ($Q_{eff}=\kappa \cdot A\cdot \frac{\Delta T}{L_{eff}}$, where,$L_{eff}=effective\;heat\;transfer\;distance,A=heat\;transfer\;area$), PPM enhances net heat delivery by: (i) Shortening conduction paths (Leff↓): Sublimation occurs at distributed sites, reducing the distance heat must travel; (ii) Expanding heat transfer area (A↑): Pore walls provide additional solid-phase conduits for radial conduction; (iii) Maintaining favorable ΔT: Efficient vapor removal prevents pressure-induced suppression of sublimation temperature [60]. Thus, despite lower κ, the Qeff increases as confirmed by 20–50% faster drying rates [61]. The macroscopic effect is that the equivalent heat transfer resistance is reduced. Two-dimensional simulations by Vasheghani et al. showed that in porous frozen layers, although heat flux lines are deflected around the pores, they eventually reconverge, resulting in more uniform heat distribution and eliminating the pronounced thermal bottlenecks typically observed in dense frozen solids [62]. Therefore, by modifying sublimation dynamics and diversifying heat transfer pathways, PPM effectively disperses thermal resistance and mitigates heat transfer limitations at the system level. Although pores decrease the microscopic solid-phase conductivity, macroscopic heat delivery is improved in practice: simulation indicates that, in samples with initial porosity, the rate of increase in dry-layer thickness is ~25% lower than in non-porous samples, consistent with a shorter effective conduction path (Leff) [63]. In addition, 2-D simulations showed that heat-flux lines, while deflected by pores, reconverge to yield a more uniform temperature distribution, eliminating pronounced thermal bottlenecks typical of dense frozen solids [62]. From an operational perspective, a 20–40% time reduction translates into a ~25–67% throughput increase for a fixed dryer (batches per unit time scale approximately with 1/(1 − ηt), where ηt is the fractional time reduction). Assuming energy consumption scales mainly with drying time, the same reduction implies a proportional energy saving. For illustration, if a batch removes 5000 kg of water and the plant’s specific energy consumption is ≈1.2 kWh kg^−1^ water removed, a 20–30% reduction corresponds to 1200–1800 kWh saved per batch; at typical industrial electricity rates, this yields tangible cost savings and capacity gains. Actual values will depend on product load, utility tariffs, and equipment efficiency.

#### 3.1.3. Reduced Dry Layer Thickness Effect

The presence of pores also effectively reduces the apparent thickness of the dried layer. Because sublimation can occur simultaneously within the internal pore space, the drying process does not rely solely on a continuous advancement of the sublimation front across the entire material. Instead, multiple regions can dry concurrently, which decreases the average thickness through which heat and mass must be transferred. For example, simulation studies have shown that in samples containing initial porosity, the rate of increase in dry layer thickness over time is approximately 25% lower than in non-porous samples, as the regions surrounding the pores quickly become fully dried and no longer contribute to the effective resistance of the layer [63]. This mitigates the progressive rise in drying resistance typically observed during the latter stages of freeze drying. Conceptually, the porous medium functions like a network of “holes” or “channels” within the dry layer, transforming it from a continuously thickening, solid barrier into a mesh-like or sieve-like structure. As a result, the average thickness and density of the resistance barrier are reduced, facilitating more efficient heat and mass transfer. This phenomenon constitutes a key mechanism of resistance reduction enabled by porous media.

**Figure 3:**
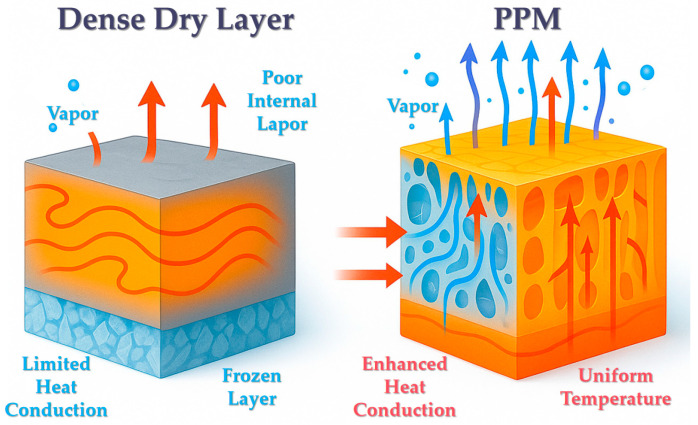
Mechanism of influence of preformed porous medium on heat and mass transfer resistance in vacuum freeze-drying. (This original conceptual figure was created by the authors using 3D Max to generate the base 3D schematic elements, followed by refinement, labeling, and layout adjustments in Adobe Illustrator 2024. The figure is illustrative only and not derived from numerical simulation data.).

In summary, PPM significantly reduces heat and mass transfer resistance during freeze drying through multiple synergistic mechanisms, including increased porosity, formation of continuous vapor escape channels, dispersion of the sublimation front, and reduction of the effective dry layer thickness. This resistance-mitigation effect lies at the core of the PPM enhancement mechanism and enables a marked increase in drying rate even in the absence of external energy fields. However, it is important to note that porosity and pore size must be carefully optimized. Excessively high porosity can drastically reduce thermal conductivity and may inadvertently prolong the drying process, while overly large pores may compromise the structural integrity of the final product. Therefore, the pore architecture must be finely tuned in practical applications to achieve an optimal balance between transport efficiency and product quality. The mechanism of influence of preformed porous medium on heat and mass transfer resistance in vacuum freeze-drying is shown in Figure 3.

### 3.2. Promotion of Convection and Uniform Heat Transfer (Internal Convection and Fluidization Effects)

In conventional freeze-drying processes, the presence of high vacuum conditions essentially eliminates convective heat and mass transfer, which not only limits overall thermal transfer efficiency but also leads to non-uniform drying across the sample (e.g., faster drying at the periphery due to increased radiative exposure from chamber walls) [64]. The introduction of preformed porous media (PPM), under appropriate conditions, can induce or enhance localized convective flow, thereby improving both the uniformity and rate of heat and mass transfer.

#### 3.2.1. Internal Gas Flow

When PPM contains macropores (>100 μm) and chamber pressure is moderately elevated (e.g., 10–100 Pa), bulk flow of water vapor may supplement diffusion-based transport. This occurs because water vapor partial pressure dominates the gas phase (≈95–99% of total pressure), and non-condensable gases (e.g., residual air) contribute minimally (<5%) under optimized vacuum conditions [23,65]. Such bulk vapor flow—governed by viscous (Poiseuille) mechanisms—enhances both vapor removal and convective heat transfer to sublimation fronts, distinct from pure diffusion [66]. These mechanisms introduce a weak form of convection that can accelerate the removal of sublimated vapor from pore surfaces and facilitate inward transport of warmer external gas, which enhances heat transfer to the ice at the pore walls. Although convection is typically negligible in conventional vacuum freeze drying, the presence of large and connected pores in PPM provides potential pathways for localized gas movement. Several researchers have employed generalized diffusion models such as the Dusty-Gas Model (DGM) to capture these effects. In porous dried layers with large pores, models incorporating convection terms (e.g., DGM) have been shown to fit experimental drying curves more accurately than pure Knudsen diffusion models [65]. For instance, a 2022 study by Levin et al. comparing Knudsen and Dusty-Gas Models in the simulation of freeze drying in bulk food materials found that the DGM-predicted drying rates were slightly higher and more consistent with experimental results, while Knudsen-based predictions underestimated the rates [66]. These findings suggest that in loosely structured, highly porous food matrices, weak internal convection may play a supporting role in mass transfer. Accordingly, by forming large, continuous pores, PPM may partially restore internal gas mobility and thereby enhance drying efficiency.

#### 3.2.2. Solid Particle Fluidization

In addition to porosity inherent in the material itself, certain process designs incorporate inert solid particles mixed with the product to form a “powder–particle system,” which can exhibit behavior analogous to a fluidized bed. A representative example is the spray freeze-drying system with inert particle fluidization, in which atomized droplets freeze upon contact with fluidized cryogenic particles, forming thin films. During drying, the inert particles remain in a fluidized state, while dried powders gradually detach and mix into the particle bed, creating a dynamic mixture of powders and particles. Although the entire process operates under vacuum, the relative motion and collisions between particles simulate a form of macroscopic convective circulation within the bed. On one hand, particle turnover helps continuously expose internal, partially dried material to the bed surface, reducing the risk of localized mass transfer dead zones. On the other hand, particle–powder collisions generate localized frictional heat and mechanically disrupt the dried layer, thereby enhancing both heat and mass transfer. This phenomenon, referred to as mechanical fluidization, effectively compensates for the lack of convective gas flow in vacuum conditions. Studies on such “powder–particle fluidized bed freeze drying” have shown that, compared to static freeze-drying, this approach can reduce drying time by more than 20% and significantly improve drying uniformity [67]. Broadly speaking, the porous media concept can be extended to include such particle-fluidized systems, in which mechanical agitation contributes to reduced transport resistance and enhanced thermal and mass transfer performance.

#### 3.2.3. Uniform Drying and Mitigation of Edge Effects

Porous structures can also help mitigate non-uniformity in the freeze-drying process. A common issue in freeze-dried foods is the edge–center drying imbalance, where peripheral regions dry faster than the core due to greater exposure to radiative heat from the chamber walls. After the introduction of PPM, the drying rate in the central region tends to increase more, reducing the difference from the edge, because the internal pores make drying easier. [68]. For example, in kiwi slice freeze-drying experiments, untreated samples showed a 4-h delay in center drying compared to the edge. In contrast, samples pretreated by mechanical perforation (to create internal pores) reduced this drying lag to less than one hour, indicating improved synchronization. In addition, the presence of macropores is also conducive to the release of water vapor pressure during the drying process, avoiding the ‘drying lag’ caused by local high pressure in the central region [69]. Thus, PPM contributes to more uniform moisture removal across the entire sample. This effect is particularly valuable for large-sized products—such as whole fruits, vegetables, or meat blocks—where the risk of incomplete drying or central quality degradation is significant. PPM-based strategies offer a promising solution for improving overall drying homogeneity and ensuring product integrity.

**Figure 4:**
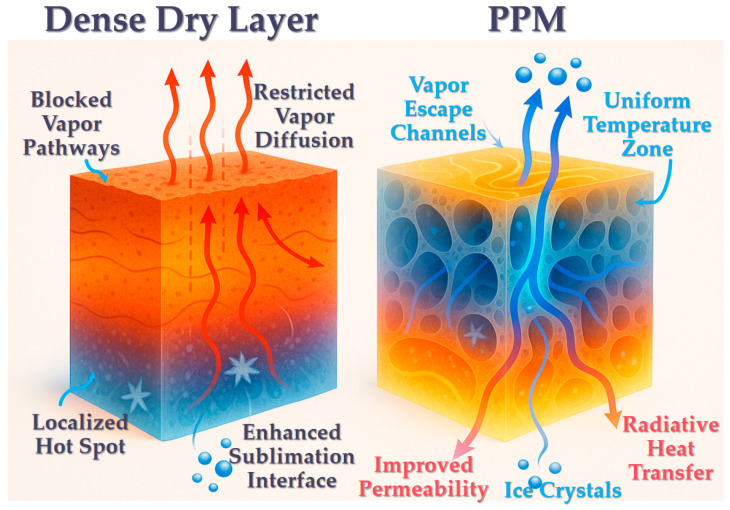
Promoting mechanism of preformed porous medium on convection and uniform heat transfer in vacuum freeze-drying process. (This original conceptual figure was created by the authors using 3D Max to generate the base 3D schematic elements, followed by refinement, labeling, and layout adjustments in Adobe Illustrator 2024. The figure is illustrative only and not derived from numerical simulation data).

It is important to note that under vacuum conditions, true gas-phase convection remains inherently weak. Even with the introduction of pores or particles via PPM, the system cannot achieve the level of convective heat transfer seen in atmospheric drying. However, PPM-induced “pseudo-convective” effects manifest on both microscopic and macroscopic levels. At the microscale, mechanisms such as slip flow and entrance effects within the pore channels enhance vapor mobility. At the macroscale, particle movement, localized thermal disturbances, and surface agitation mimic convective heat transfer, thereby accelerating drying and improving uniformity. If PPM-based strategies are combined with moderate vacuum levels (i.e., non-extreme vacuum) or cyclic pressure variations, these convective-like effects may be further amplified. Preliminary investigations—such as pulsating VFD—have already demonstrated the potential to shorten drying times under such conditions. In essence, PPM introduces dynamic elements into an otherwise static freeze-drying system, making it more akin to a fluidized or convective drying regime. This shift results in more efficient and uniform heat and mass transfer performance overall [70]. The promoting mechanism of preformed porous medium on convection and uniform heat transfer in the vacuum freeze-drying process is as follows in Figure 4.

### 3.3. Enhancement of Radiative Heating (Porous Absorption and Transmission Effects)

Radiative heat transfer is one of the primary modes of energy input during freeze drying, with infrared radiation emitted from the shelves and chamber walls directly heating the material surface and partially penetrating into its interior [71]. The presence of preformed porous media (PPM) can influence the way radiation is transferred and absorbed, potentially enhancing its effectiveness and enabling more efficient utilization of radiative energy.

#### 3.3.1. Improving Radiative Absorptivity

For materials with light color or smooth surfaces, the proportion of incident thermal radiation that is directly absorbed is often limited. In contrast, porous structures typically exhibit high specific surface area and multiple scattering effects, which can enhance the capture and absorption of radiative energy [72]. The internal pore walls and cavities function as micro-resonant chambers, where incoming infrared radiation undergoes multiple reflections, increasing the likelihood of absorption by the pore wall material and thereby effectively raising the overall absorptivity. Studies have shown that coating freeze-dried samples with black porous carbon materials significantly increases radiative absorption efficiency and accelerates the drying rate (PPM increases radiative absorptivity (α) by 40–60% compared to non-porous surfaces. Carbon-coated freeze-dried samples exhibit α = 0.85–0.92 vs. 0.55–0.65 for uncoated controls) [73,74]. This demonstrates the potential of radiation-absorbing porous media to improve thermal energy uptake. Similarly, when the material itself forms a roughened, porous surface, it can enhance internal reflection and absorb more radiative heat. This principle is especially applicable in microwave-assisted freeze drying, where the addition of radiation-absorbing porous materials—such as conductive carbon black—can increase microwave absorption and convert it more efficiently into heat, resulting in more uniform internal heating [75]. In one study, Wang et al. added porous dielectric particles with high loss factors to vitamin C solutions and observed over a 70% reduction in energy consumption due to improved microwave heating efficiency. These findings illustrate that porous media can, in some cases, serve dual functions—as structural scaffolds and wave absorbers—by extending optical path lengths and enhancing radiative energy capture to improve the efficiency of heat delivery during drying [76].

#### 3.3.2. Increasing Radiative Penetration Depth

**Figure 5:**
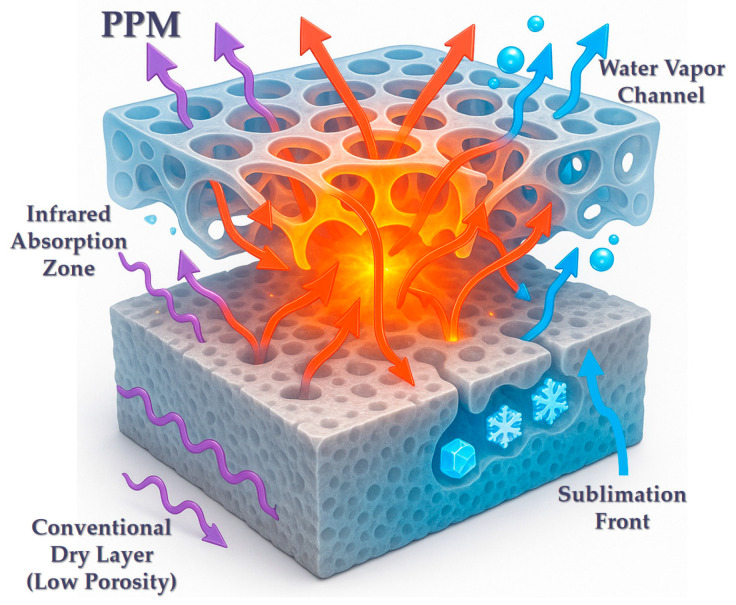
Radiation enhancement mechanism of preformed porous medium for vacuum freeze-drying (porosity absorption and transmission effect). (This original conceptual figure was created by the authors using 3D Max to generate the base 3D schematic elements, followed by refinement, labeling, and layout adjustments in Adobe Illustrator 2024. Color legend: Purple (wavy) arrows denote incident and transmitted infrared/electromagnetic radiation within the porous layer (porous absorption and transmission); Red/Orange arrows indicate secondary heat redistribution and conduction from the radiative absorption zone; Blue arrows represent water vapor escape pathways and mass transfer from the sublimation front. Snowflake icons mark the frozen region. This is an original conceptual schematic (authors), created for mechanism visualization (no numerical field data).

For a given wavelength of radiation, the effective penetration depth into a material determines how deeply thermal energy can be delivered. In dense solids, infrared radiation may only penetrate a few microns, whereas the presence of pores allows radiation to propagate further along pore channels. Within a porous dried layer, infrared light can travel deeper than in a solid matrix, as the voids act as quasi-transparent pathways for radiation [72]. This enables thermal energy to reach regions closer to the sublimation front, reducing surface overheating and minimizing energy loss due to accumulation near the exterior. Simulations by Li et al. demonstrated that in near-infrared drying of pomegranate samples, increasing porosity led to more uniform internal temperature profiles and faster predicted drying rates, suggesting that porous structures improve the internal distribution of radiative heat (PPM doubles radiation penetration depth δ: IR drying of pomegranate: δ = 2.5 mm in porous samples (50% porosity) vs. 0.8 mm in non-porous) [76]. Furthermore, microwaves, as a form of electromagnetic radiation, also exhibit penetration depths that depend on the material’s dielectric properties and porosity. Since dried layers with low moisture content absorb microwaves poorly, pores can act as conduits that allow microwaves to reach the still-frozen regions beneath the dried crust, thereby enabling direct heating at the sublimation interface. In a study by Gruber et al., freeze-dried samples with preformed pores exhibited deeper microwave field distribution and faster temperature rise at the sublimation front (Microwaves penetrate >15 mm in porous enzyme samples (ϕ = 70%) vs. 5 mm in controls, enabling direct heating of sublimation fronts) [77]. These findings indicate that porous media can function as thermal waveguides or lenses, directing radiative energy deeper into the material and improving heating efficiency during the drying process. The radiation enhancement mechanism of preformed porous medium for vacuum freeze-drying (porosity absorption and transmission effect) is shown in Figure 5.

In summary, preformed porous media (PPM) enhance radiative heat transfer by altering the material’s electromagnetic absorption and transmission characteristics. On one hand, porous structures increase radiation capture by reducing surface reflectance losses; on the other, they facilitate deeper and more uniform distribution of radiative energy within the material. This effect is particularly pronounced when infrared or microwave-assisted freeze drying is applied. However, it is important to note that radiative enhancement can also introduce the risk of localized overheating. Therefore, the pore architecture and radiation parameters must be co-optimized to ensure that product quality is not compromised. Overall, PPM provides a novel, passive energy-field intensification strategy for more effective utilization of radiative heating during freeze drying. The relative contribution of different enhancement mechanisms varies across food systems and pore structure designs. In large, thick samples, resistance reduction and thermal conduction optimization are more dominant, whereas in thin-layer materials, radiative and convective enhancements may play a greater role. In most cases, only the overall change in drying rate is experimentally observable, making it necessary to employ modeling tools and advanced diagnostics—such as cryo-CT scanning [78] or infrared thermography—to validate the underlying mechanisms. Consequently, elucidating the mechanism-specific roles of porous media under varying conditions remains a critical area of academic interest and a foundational step toward improving process reliability.

## 4. Research Progress on Freeze-Drying Strengthening of Liquid Food and Solid Agricultural Products

### 4.1. Freeze-Drying Strengthening of High Value-Added Liquid Food

#### 4.1.1. Dairy Products

Freeze drying of dairy products is widely used in the production of milk powders and high-end infant formulas. However, conventional tray freeze drying of milk liquids is often limited by high viscosity and concentration, resulting in long drying times and the formation of hard surface crusts. In recent years, preformed porous structures and structural modification strategies have been introduced to significantly improve freeze-drying performance for dairy systems [79]. For example, Wang et al. applied a spray freezing pretreatment to mare’s milk by atomizing the liquid into ~50 µm droplets and freezing them in cold air to form porous frozen granules, which were then freeze-dried. Compared to direct tray drying, this approach reduced drying time by approximately 35%, while maintaining good solubility and nutrient retention in the final milk powder [80]. The enhancement is attributed to the increased specific surface area of microdroplets and the presence of interparticle voids, which greatly shorten the sublimation path. In another study on cow’s milk, a whipping–foaming step was introduced: milk was vigorously whipped with a small amount of emulsifier to form a stable foam before freezing. The resulting freeze-dried foam powder exhibited a porous internal structure, a 25% reduction in drying time, and improved powder looseness and rehydration [81]. Additionally, incorporating small amounts of proteins as ice nucleating agents in dairy matrices helped refine ice crystal morphology, promote pore formation, and improve both drying rates and powder structure [82]. From a modeling perspective, Shao et al. used a cellular automaton approach to simulate the foam freeze-drying process of carotenoid-loaded emulsions, showing that models incorporating preformed pores provided more accurate predictions of drying kinetics—highlighting the need to reflect structural parameters in simulation frameworks [83]. In summary, for dairy and other liquid food systems, preformed porous media strategies—such as spraying and foaming—have been successfully applied in experimental studies, significantly improving freeze-drying efficiency without compromising product quality. These methods offer promising guidance for developing more efficient industrial-scale milk powder freeze-drying processes.

#### 4.1.2. Coffee, Tea, and Beverage Extract

Instant coffee is a classical application of freeze-drying technology. However, the high concentration and viscosity of coffee extracts result in slow freeze-drying rates, while traditional spray drying compromises aroma quality. Consequently, improving the efficiency of coffee freeze drying has long been a subject of interest. Preformed porous media (PPM) strategies have also demonstrated strong potential in this context. Ghamdi et al. applied a vacuum foaming pretreatment to coffee extract, where reduced pressure induced the release of dissolved gases, creating a foam structure. The foamed coffee was then freeze-dried using microwave-assisted heating, resulting in over 40% reduction in drying time while preserving aroma and achieving excellent instant solubility [29]. The preformed pores provided by foaming facilitated rapid vapor escape under microwave irradiation, while the foam structure mitigated the risk of localized overheating due to dielectric non-uniformity. In another study, Zhang et al. incorporated wave-absorbing nanoparticles (e.g., Fe_3_O_4_) into coffee extract and applied spray freezing. The resulting microporous matrix exhibited rapid internal heating under microwave exposure, reducing drying time by nearly 50% [84]. These findings underscore the synergistic effect of combining PPM with energy fields, enabling significant process intensification without sacrificing aroma quality. Similar approaches have been explored in tea infusions and fruit juice concentrates. For example, converting concentrated juice into foams or gel beads with internal porosity prior to freeze drying helps prevent crust formation and promotes uniform drying. Studies have shown that foamed orange juice powders exhibit a 15% increase in porosity and improved vitamin C retention compared to controls [85]. Overall, for high-value beverages that require maximum aroma and bioactivity preservation, preformed porous strategies not only enhance drying efficiency but also help retain flavor, since faster drying minimizes volatile compound loss. Some premium instant beverage manufacturers have begun adopting foam-assisted freeze-drying to improve product quality and reduce energy consumption [86].

#### 4.1.3. Fermentation Broth and Bioactive Liquid

Freeze-drying of yeast cultures, microbial suspensions, and enzyme solutions is traditionally employed for the production of microbial powders or enzyme preparations [87,88]. These systems typically contain high concentrations of proteins and polysaccharides, which tend to form gel-like matrices during freezing, making drying difficult and increasing the risk of biological inactivation. Recent studies have explored the use of preformed porous structures to alleviate these challenges. For example, in the freeze-drying of lactic acid bacteria fermentation broth, Ciurzyńska et al. introduced pore-forming protectants such as sucrose and alginate and employed a staged freezing protocol. The resulting porous bacterial powders exhibited over 20% higher viability and approximately 15% shorter drying times compared to non-porous controls, likely due to reduced mechanical stress and enhanced moisture diffusion enabled by the internal pore network [89]. Similarly, enzyme solutions subjected to vacuum freeze concentration with concurrent stirring formed aggregated ice structures that left behind porous networks. Freeze-dried samples with this structure retained over 90% enzymatic activity, whereas samples without pores, exposed to longer heat exposure, showed activity loss down to 70%, indicating that rapid water removal helped mitigate thermal denaturation [90]. In addition, porous structures facilitate rapid rehydration of bio-powders. For example, effervescent tablet-type enzyme formulations are often prepared via porous freeze-drying, allowing for immediate disintegration and enzyme release upon contact with water [91]. Overall, in biotechnological systems such as fermentation broths, PPM strategies offer a promising dual benefit—accelerated drying and enhanced bioactivity preservation. Experimental results to date have been encouraging, although system-specific optimization is required (e.g., via foaming, gelation, or carrier embedding). These approaches hold strong potential for the development of tailored freeze-drying protocols for different microbial and enzymatic solutions.

### 4.2. Freeze-Drying Strengthening of Solid Agricultural Products

#### 4.2.1. Vegetable Fruits

Fruits and vegetables, rich in vitamins and pigments, represent a major category in freeze-drying applications. However, their complex tissue structure—including rigid cell walls and protective skins—often imposes resistance to mass transfer during drying [92]. For example, intact-skinned fruits such as blueberries and grapes exhibit slow drying rates and are prone to uneven moisture distribution, with dry exteriors and moist interiors [93,94]. To address this, mechanical perforation or surface etching is a direct preformed porous media (PPM) strategy. Munoz et al. reported the use of CO_2_ laser micro-drilling to create an array of ~100 µm pores (~50 pores per fruit) on the blueberry surface. This enabled vapor to escape primarily through the micro-channels, reducing the total drying time from 30 to 20 h while ensuring uniform dehydration without internal collapse. Sensory evaluations indicated no significant difference in color or nutritional quality between laser-treated and untreated samples [95]. Similarly, for waxy-skinned fruits like grapes or jujubes, manual puncturing or peeling can significantly accelerate drying [96]. Another approach involves ice crystal engineering during pre-freezing: by adjusting cooling rates or introducing annealing steps, large ice crystals can form and leave behind macro-scale pores. For instance, Sappati et al. applied staged freezing to kelp, where slower freezing yielded larger ice crystals and, after drying, a macroporous structure that facilitated moisture release and shortened drying time by 15% [97]. However, excessive pore size may damage tissue integrity, requiring careful control. For sliced fruits and vegetables (e.g., carrots, apples), pulsed electric field (PEF) or high hydrostatic pressure (HHP) pretreatments are commonly used to induce electroporation or partial cell wall disruption, forming functional micropores [98]. Zhang et al. showed that HHP pretreatment (400 MPa) of strawberries before vacuum freeze-drying increased tissue porosity, reduced drying time by 18%, and improved rehydration performance [99]. Similarly, ultrasound pretreatment can generate micro-channels and expel some water, facilitating subsequent drying. For example, red bell pepper slices subjected to 5 min of high-power ultrasound developed internal cracks and pores, resulting in a 20% reduction in drying time and improved vitamin C retention [100]. These methods essentially use external fields to introduce or enlarge porosity in solid plant tissues, thereby reducing mass transfer resistance—an approach that fits broadly within the PPM framework. On the modeling side, Bogusz et al. developed a spectral method to simulate freeze drying of kiwifruit, incorporating pore distribution parameters, which improved agreement with experimental heat and mass transfer behavior [101]. This highlights the importance of structure-aware modeling for non-homogeneous food matrices such as fruits and vegetables.

#### 4.2.2. Medicinal and Spice Plants

Freeze drying has long been used for the preservation of medicinal and aromatic plants, such as ginseng, goji berries, mushrooms, and edible flowers [102]. To improve drying efficiency for these high-value solid agricultural products, researchers have explored various structural enhancement strategies. For example, ginseng, known for its dense tissue structure, is challenging to freeze-dry directly due to prolonged drying times and risk of internal discoloration. Lee et al. addressed this by embedding fresh ginseng in an alginate hydrogel, freezing it into porous gel blocks prior to freeze-drying. This approach accelerated drying and increased the retention of ginsenosides by 15%, likely due to the gel network providing additional vapor escape channels and more uniform heat transfer, while minimizing structural collapse of the ginseng itself [103]. For sliced or powdered herbal materials, freeze-induced expansion methods have been applied. Zhang et al. demonstrated that freeze–thaw cycling of goji berries generated microcracks caused by ice crystal expansion, leading to the formation of internal pores. This enhanced the subsequent drying rate and improved the yield of goji polysaccharides, presumably due to the increased effective drying and extraction surface area [104]. In some cases, enzymatic pretreatment or fermentation is used to soften the cell wall structure before drying, effectively generating internal pore networks. For aromatic herbs such as mint or basil, studies have shown that vacuum–microwave hybrid pre-drying can induce micro-explosive pore formation in leaf tissues, which accelerates freeze drying while preserving volatile aroma compounds [105,106]. Overall, for medicinal and aromatic plants, PPM techniques are often coupled with quality preservation strategies. By creating controlled pore structures, these methods not only reduce drying time but also lower thermal exposure, thereby improving the retention of bioactive and flavor compounds. This dual benefit helps explain the growing interest in PPM approaches for high-value botanical applications.

#### 4.2.3. Aquatics and Meat

Freeze-dried seafood and meat products are known for their ability to retain maximum nutritional and flavor qualities, yet they often suffer from prolonged drying times. This limitation is primarily due to the high protein content in such matrices—especially in fish muscle—which forms a continuous frozen network that hinders sublimation due to limited mass transfer pathways [107]. One strategy to address this is air bubbling pretreatment prior to freezing. For instance, inert gas can be introduced into minced fish slurry to form a foam structure, which is then freeze-dried into a porous fish powder. This method reduces drying time to approximately 20 min, achieving several-fold acceleration compared to non-foamed controls, while yielding a loose porous texture with excellent rehydration characteristics [108]. For large meat blocks, a composite technique combining structural support and microwave-assisted drying has been proposed. Specifically, porous ceramic rods are inserted into the meat to act as heat-conductive scaffolds, while microwave irradiation provides internal heating. This synergistic setup significantly reduces drying time [109]. Related work by Wu et al. has explored fluidized bed freeze-drying of seaweed (as previously discussed), and similar approaches have been investigated for small shrimp, wherein solid spherical media are used to enhance heat and mass transfer through mixed flow configurations [110]. Nonetheless, the complex flavor compounds and intricate tissue structures of seafood and meat pose challenges, and current research on PPM (Preformed Porous Media) applications in these products remains relatively limited. It is anticipated that future developments—especially those integrating energy-assisted techniques such as vacuum freeze–microwave drying, already commercialized for crispy seafood snacks—will increasingly rely on engineered pore structures as a key enabling factor for drying efficiency and product quality.

Whether applied to high-value liquid foods or solid agricultural and medicinal materials, Preformed Porous Media (PPM) technology has consistently demonstrated a broadly applicable enhancement effect on freeze-drying performance. Experimental studies unanimously show that the introduction of pore structures can reduce freeze-drying time by approximately 20–50% without compromising product quality. In most cases, product attributes are even improved, owing to the milder drying conditions enabled by enhanced mass and heat transfer through the porous matrix. A summary of the applications of preformed porous media (PPM) in food freeze-drying is given in Table 3.

## 5. Discussion and Outlook

### 5.1. Comparative Discussion

Evidence across product types indicates that PPM effectiveness is primarily governed by pore connectivity and size: interconnected macropores > closed/isolated pores > non-porous matrices in terms of drying rate, due to reduced diffusion resistance and distributed sublimation fronts (Section 3.1). Representative cases compiled in Table 3 show ~20–50% time reductions together with quality benefits (instant solubility, rehydration, aroma retention) in liquids and solids, e.g., coffee (vacuum foaming + microwave): >40%, Fe_3_O_4_-assisted spray-freezing coffee extract: ~50%, blueberries: 30→20 h. These trends are consistent with modelling and experimental observations that larger, continuous pores increase effective diffusion coefficients and lower vapor-pressure gradients, thereby accelerating sublimation (Section 3.1). Moreover, porous architectures redistribute heat flux and improve temperature uniformity, alleviating bottlenecks typical of dense matrices (Section 3.1.2).

### 5.2. Core Challenges

Despite the significant potential of Preformed Porous Media (PPM) technology in vacuum freeze-drying of agricultural and food products, numerous critical challenges remain to be addressed. Foremost among these is the precise regulation of pore structures. Current research has yet to fully elucidate the mechanistic relationships between pore characteristics and drying performance, making it difficult in practice to achieve high-level synergy and accurate control between structural attributes and functional outcomes. In particular, the precise manipulation of key structural parameters, such as pore size distribution, porosity, and interconnectivity, remains technologically constrained. These limitations highlight the urgent need for fundamental studies and innovative technological advancements aimed at overcoming these bottlenecks and enabling more reliable control of internal porous architectures [110]. While PPM strategies exhibit wide applicability, their implementation must be product-specifically optimized. Key determinants include initial moisture content, structural integrity, and sensitivity to pore-forming treatments. For example, low-moisture/high-lipid matrices (e.g., oilseeds) may require hybrid approaches combining mild mechanical poration with antioxidant infusion to mitigate quality risks. Ongoing advances in precision pore engineering will further broaden PPM adaptability.

Secondly, the design and fabrication of gradient pore structures remain insufficiently developed both theoretically and practically. There is currently a lack of established theoretical frameworks and empirical methodologies to guide the rational design of gradient porous architectures tailored to the specific requirements of different agricultural and food products. In particular, the ability to engineer spatial variations in pore size and distribution according to functional needs is still largely underexplored. Moreover, the scalable manufacturing of gradient structures is hindered by unresolved technical challenges, including complex equipment requirements, high production costs, and process instability. These limitations significantly constrain the broader industrial implementation of PPM technology and underscore the need for dedicated research and innovation in controllable and cost-effective gradient pore fabrication strategies [111].

Thirdly, the application of multi-technology coupled enhancement strategies remains in its early exploratory phase. The synergistic mechanisms underlying the integration of PPM technology with auxiliary energy fields—such as microwave, infrared, ultrasound, and other intelligent control techniques—have yet to be clearly elucidated. In particular, the complex interplay of multi-factor influences under coupled heat and mass transfer processes requires further in-depth investigation. At present, there is a lack of comprehensive and predictive optimization models capable of guiding real-world industrial implementation. This limitation hampers the advancement and widespread adoption of synergistic enhancement technologies in vacuum freeze-drying applications [112].

### 5.3. Engineering and Economic Implications

For a fixed dryer, a 20–40% reduction in drying time implies a ~25–67% increase in throughput (batches per time ∝ 1/(1 − η_t_), η_t_ = fractional time saving). Assuming energy scales with time, this also translates to proportional energy savings. For illustration, removing 5000 kg of water at a plant-level SEC of ≈1.2 kWh kg^−1^ water removed, a 20–30% reduction saves 1200–1800 kWh per batch. These gains, combined with improved rehydration/quality reported in Table 3, underscore PPM’s practicality for scale-up. Actual savings depend on load, tariff, equipment efficiency, and product-specific structural targets.

### 5.4. Future Outlook

In response to the aforementioned challenges, future development of preformed porous media (PPM) technology should emphasize interdisciplinary integration and the application of innovative enabling technologies. First, advanced manufacturing techniques such as 3D printing and micro/nano-fabrication will become critical tools for the precise construction of porous structures. These technologies offer enhanced flexibility, controllability, and reproducibility, thereby facilitating truly customized pore architecture design [113,114]. Second, the implementation of multiscale modeling and simulation will significantly improve our understanding of heat and mass transfer mechanisms during freeze-drying, enabling accurate prediction and process optimization [115]. Meanwhile, the development of intelligent online monitoring and feedback control systems can achieve real-time tracking and autonomous regulation of the drying process, further enhancing its efficiency, consistency, and reliability [116].

At the industrial level, the development and deployment of continuous and large-scale production equipment will be a crucial pathway for advancing preformed porous media (PPM) technology from laboratory research to industrial-scale implementation. This transition requires not only iterative optimization of technical equipment, but also the establishment of comprehensive standards and quality control protocols tailored to industrial needs, in order to ensure the stability, scalability, and sustainability of PPM applications [117]. Through multidimensional and systematic innovation, PPM technology is expected to achieve broader and deeper integration into agricultural and food processing sectors, significantly enhancing the overall technological level and global competitiveness of the industry. Ultimately, this progression will drive the shift from empirical trial-and-error approaches toward model-driven process engineering, enabling the transformation of agri-food freeze-drying toward greater efficiency, low-carbon footprint, and intelligent control [118].

## 6. Conclusions

Preformed porous media (PPM) provide a structure-based pathway to mitigate both heat and mass-transfer resistances in vacuum freeze-drying (VFD). By creating open and continuous pore networks—often via spray-based routes such as SFD—PPM shortens effective dry-layer thickness, disperses the sublimation front, and establishes low-resistance vapor channels. Across diverse agri-food systems, PPM consistently reduces total drying time by ~20–50% with quality benefits (e.g., instant solubility, rehydration, aroma retention), as evidenced by representative cases compiled in this review (e.g., coffee with vacuum foaming + microwave: >40%, Fe_3_O_4_-assisted spray-freezing coffee extract: ~50%, laser-micro-drilled blueberries: 30→20 h) [84,85,96]. These outcomes align with mechanistic evidence that larger, connected pores increase effective diffusion coefficients and lower vapor-pressure gradients, thereby accelerating sublimation [57,58,59,60,61,62,63]. From an engineering standpoint, SFD-enabled PPM emerges as a practical, material-intrinsic intensification strategy that is complementary to field-assisted methods and amenable to integration in liquid and solid matrices [47,48,49,50,51,52,53,54]. Remaining limitations primarily concern precise structural control, scalable fabrication (including gradients), and coupling with auxiliary energy fields—gaps that shape us to invest more energy in future research to achieve, and inject strong innovation momentum into the discipline of food engineering and agricultural product processing.

## Figures and Tables

**Table 1:** Main Types and Comparison of Programmable Spray Technologies for Porous Structure Formation.

Spray Freezing Type	Freezing Medium	Freezing Mechanism	Product Morphology	Advantages	Limitations	Suitable Applications	Ref.
SFV/L	Vapor layer above cryogenic liquid (e.g., N_2_)	Droplets pre-cooled in cold vapor, then solidified upon contact with cryogenic liquid	Microparticles	Reduces droplet splashing; easier particle collection	Partial freezing before immersion; potential droplet coalescence; cryogen recovery needed	Powdered nutraceuticals, flavors	[39]
SFV	Cold gas (e.g., refrigerated N_2_, scCO_2_)	Droplets gradually freeze while suspended in cold vapor flow	Irregular particles or aggregates	Simplified equipment; avoids cryogenic liquid handling	Incomplete freezing; particle shape variation due to short vapor residence time	Sensitive compounds, volatile aroma ingredients	[40]
SFL	Liquid nitrogen or other cryogenic liquids	Droplets rapidly freeze upon direct contact with cryogen	Fine porous microspheres	Highest freezing rate; fine ice crystals and high porosity	Nozzle blockage risk; high-pressure required; safety and complexity concerns	Dairy powders, probiotic formulations, inhalable particles	[41]
SFCS	Pre-cooled metal plate (solid surface)	Droplets flatten, freeze into thin films on contact with cold plate	Porous flakes or sheets	Uniform film freezing; suitable for viscous or high-sugar formulations	Requires scraping and transfer step; limited particle control	Instant beverages, fruit juice sheets, sugar-rich food extracts	[43]

**Table 2:** Summary of the relationship between process parameter control and pore structure regulation in spray freeze drying.

Process Parameter	Control Strategy	Effect on Pore Structure	Practical Considerations	Ref.
Droplet Size	Select nozzle type to adjust atomization (pressure, pneumatic, ultrasonic)	Small droplets (1–50 μm): 0.5–5 μm pores, 70–90% porosity Large droplets (200–1000 μm): 20–100 μm pores, 40–60% porosity	Align droplet size with desired pore size; avoid broad distribution	[47]
Initial Concentration	Adjust solid content in feed solution	Low conc. (5–10%): 50–200 μm pores, 80–95% porosity Medium conc. (10–30%): 20–50 μm pores, 60–80% porosity High conc. (>30%): <20 μm pores, 30–50% porosity	Balance porosity with mechanical strength	[48]
Freezing Medium Temperature	Use cryogens like liquid nitrogen vs cold gas	Liquid N_2_ (−196 °C): 0.1–5 μm pores, 85–95% porosity Cold gas (−40 °C): 10–100 μm pores, 50–70% porosity	Cryogen consumption and safety	[49]
Freezing Rate	Control cryogen flow rate and exposure time	Fast (>10^3^ K/s): 0.1–1 μm pores, 85–95% porosity Slow (~10^1^ K/s): 10–100 μm pores, 50–70% porosity	Avoid over-fast freezing that may reduce rehydration ability	[50]
Additives (e.g., Nucleating Agents)	Incorporate microparticles as ice nucleation sites	With agents (0.1–1.0 wt%): Pore size ↓30–50%, porosity ↑15–25% (e.g., 60% → 75–85%)	Ensure compatibility with feed material	[51]
Foaming Agents	Add foaming agents to introduce gas bubbles	Gas-derived pores: 50–500 μm, overall porosity 75–90%	Foam stability during freezing must be controlled	[52]
Thermal Gradient Control	Stepwise temperature reduction during spraying	Gradient pores: Surface (10–30 μm, 60–70% porosity) → Core (50–200 μm, 80–90% porosity)	Requires precise environmental control	[53]

**Table 3:** Summary of Applications of Preformed Porous Media (PPM) in Food Freeze-Drying.

Food Type	Method of PPM Formation	Enhancement Type/Effect	Ref.
Mare’s milk	Liquid atomized into ~50 μm droplets and frozen in cold air to form porous frozen granules	Drying time reduced by approximately 35%	[80]
Cow milk	Milk vigorously whipped with a small amount of emulsifier before freezing to form stable foam	Drying time reduced by 25%; improved powder looseness and rehydration	[81]
β-Carotene emulsion	Used cellular automata to simulate foam freeze-drying of carotenoid-loaded emulsions	Models incorporating preformed pores more accurately predicted drying kinetics, highlighting the necessity of structure-based modeling	[83]
Coffee	Vacuum foaming pretreatment induced bubble formation, followed by microwave-assisted freeze drying	Drying time reduced by over 40%; retained aroma and achieved excellent instant solubility	[29]
Coffee	Fe_3_O_4_ nanoparticles added to extract, then spray-frozen	Drying time reduced by nearly 50%	[84]
Juice	Concentrated juice pretreated into foam or gel beads with internal pores	Prevented crust formation and improved uniformity; foam orange powder had 15% higher porosity and better vitamin C retention	[85]
Lactic acid bacteria	Pore-forming protectants (e.g., sucrose, alginate) added, with stepwise freezing	Porous bacterial powder had over 20% higher survival rate and ~15% shorter drying time than control	[89]
Enzyme solution	Vacuum freeze-concentrated while stirring, forming porous ice network	Retained over 90% enzyme activity; non-porous sample lost activity due to prolonged heating	[90]
Blueberries	CO_2_ laser drilled ~50 micro-holes (~100 µm each) per fruit	Reduced total drying time from 30 h to 20 h; uniform dehydration without collapse	[95]
Grapes, red dates	Manually punctured or peeled	Significantly accelerated drying	[96]
Kelp	Stepwise freezing created large ice crystals and resultant macro-porous structure	Drying time reduced by ~15%; large pores promoted water release but required careful control to avoid tissue damage	[97]
Strawberries	High-pressure (HHP, 400 MPa) pretreatment increased tissue porosity	Drying time reduced by 18%; rehydration improved	[99]
Red bell pepper	Treated with 5 min high-power ultrasound to induce cracks and pores	Drying time reduced by 20%; improved vitamin C retention	[100]
Ginseng	Embedded fresh ginseng in alginate hydrogel, frozen into porous gel blocks before drying	Accelerated drying and increased ginsenoside retention by 15%	[103]
Goji berries	Freeze–thaw cycling caused microcracks from ice crystal expansion	Improved drying rate and increased polysaccharide yield	[104]
Mint, basil	Vacuum–microwave pre-drying induced micro-explosive pore formation in leaves	Accelerated drying while preserving volatile aroma compounds	[105,106]
Fish paste	Inert gas introduced to fish paste to form foam, then freeze-dried	Drying time reduced to ~20 min, several times faster than non-foamed samples; resulted in porous, fluffy texture with excellent rehydration	[108]
Chicken	Porous ceramic rods inserted for heat conduction, combined with microwave heating	Significantly reduced drying time	[109]
Kelp, shrimp	Fluidized bed freeze-drying	Used solid spherical media to enhance heat and mass transfer via mixed flow configuration	[110]

## Data Availability

No new data were created or analyzed in this study. Data sharing is not applicable to this article.
